# Relationship Between Ankle Ligamentous Injuries and Osteochondral Lesions in a Saudi Arabian Population: A Retrospective Cohort Study

**DOI:** 10.7759/cureus.69150

**Published:** 2024-09-11

**Authors:** Bashar Reda, Abdulraheem A Almokhtar, Feryal O Attiah, Lujain K Kamal, Shahad O Alshaynawi, Muath S Alghamdi, Renad Alzahrani

**Affiliations:** 1 Orthopedic Surgery, King Abdulaziz University Hospital, Jeddah, SAU; 2 Orthopedic Surgery, King Abdullah Medical City, Makkah, SAU; 3 Orthopedics, King Abdulaziz University Hospital, Jeddah, SAU; 4 Orthopedics, King Abdulaziz University Faculty of Medicine, Jeddah, SAU; 5 College of Medicine, King Abdulaziz University, Jeddah, SAU; 6 Orthopedic Surgery, King Abdulaziz University Faculty of Medicine, Jeddah, SAU

**Keywords:** ankle injury, ankle sprain, cartilage disease, osteochondral lesion, sports medicine

## Abstract

Objectives

This study aimed to investigate the relationship between ankle ligamentous injuries and osteochondral defects (OCDs) and to determine the incidence of these conditions within the Saudi Arabian population.

Methods

This retrospective cohort study included 215 patients (110 males and 105 females) who sought medical advice for an ankle injury at our institute from January 2017 to August 2022. The patients were divided according to age, sex, presence and type of ligamentous injury, and status of OCDs. Patients' ages were categorized into three groups: younger than 30 years (48 patients), 30-60 years (145 patients), and older than 60 years (22 patients). The patients were assessed for ligamentous ankle sprains, tears, and OCDs by reviewing their magnetic resonance imaging reports from radiology records. The exclusion criteria included ankle fracture, ankle surgery, osteoarthritis of the ankle joint, inflammatory arthritis, and congenital deformity of the ankle. The data were collected using a simple questionnaire created via Google Forms.

Results

Of the 24 patients with OCD, 23 had OCD of the talus. Anterior talofibular ligament (ATFL) injury was the most common injury (75 patients), whereas posterior talofibular ligament (PTFL) injury was the least common. The only significant relationship between ligamentous injury and OCD was observed between PTFL injury and OCD of the talus (P = 0.041).

Conclusions

In patients with an ankle injury, the most common OCDs occur at the talus, whereas the most injured ligament is the ATFL. Nevertheless, a significant relationship was observed between PTFL injury and OCD of the talus. The findings underscore the importance of considering specific ligament injuries when diagnosing and managing OCDs, especially in patients with chronic ankle pain.

## Introduction

In sports medicine, ankle injuries are considered a key area of interest in terms of their prevention, diagnosis, and management [1]. An ankle sprain is among the most common injuries among athletes and non-athletes [2]. During an ankle sprain, multiple ligaments can be injured depending on the mechanism of injury. The most commonly injured group of ligaments are the lateral ligaments, consisting of the anterior talofibular ligament (ATFL), calcaneofibular ligament (CFL), and posterior talofibular ligament (PTFL). Remarkably, the most common lateral ligamentous injury is an isolated ATFL injury at 70%, followed by CFL and PTFL injuries [1].

A previous study reported a grading system to classify the severity of an ankle sprain as follows: Grade I, no loss of function, negative anterior drawer test result, little or no bleeding, and no point tenderness; Grade II, some loss of function, positive anterior drawer test result, negative talar tilt test, bleeding, or point tenderness; Grade III, near total loss of function, positive anterior drawer and talar tilt test results, bleeding, extreme point tenderness, or decreased entire ankle motion [1]. There are several mechanisms of injury. The most commonly reported mechanism involves forefoot adduction, hindfoot inversion, and external tibial rotation with the ankle in plantar flexion, resulting in osteochondral defects (OCDs) of the talus and multiple ligamentous injuries [1,3-5]. OCD is defined as the separation of a fragment of the articular cartilage with or without subchondral bone, which is caused by single or multiple traumatic events involving the talar articular cartilage and its subchondral bone [6-8]. The most common OCD associated with these ligamentous injuries occurs on the talar bone and tibial plafond. These OCDs are estimated to be prevalent in 22-95% of cases involving lateral ligament injuries and often cause chronic ankle instability [9].

The diagnosis of an ankle sprain is based on medical history and physical examination, in addition to magnetic resonance imaging (MRI) and ankle arthroscopy, both of which are ideal tools for evaluating structural changes in the joint [10,11]. In the past 10 years, MRI has proven to be an invaluable modality for evaluating patients with chronic ankle pain after an injury. MRI has a superior diagnostic yield compared to other radiographic imaging modalities [12]. However, ankle sprains may be underreported, as only 50% of patients with these injuries seek medical attention [1]. Benedict DiGiovanni conducted a retrospective review of MRI examination, intraoperative findings, clinical history, and physical examinations in 61 patients and observed 15 different associated injuries; of all injuries, OCD of the talus was found in 23% of cases [3]. In 2020, Wang et al. reviewed the surgical records of 1,169 patients with a history of at least one ankle sprain and found that 37% had an OCD. A significant correlation was found between ATFL injury, CFL injury, and lateral talus OCD (p-value = 0.031 and 0.024, respectively) [13].

No recent studies among the Saudi Arabian population have examined the association between ankle ligament sprain or tear and OCDs. Therefore, this study aimed to assess the correlation between ankle ligaments and OCDs of the ankle and to determine the incidence of ligamentous injury and OCDs among adult patients treated at our institution.

## Materials and methods

Ethical statement

This study and all associated protocols were approved by the Biomedical Ethics Research Committee of King Abdulaziz University Hospital (KAUH), Jeddah, Saudi Arabia (approval number: 526-22). All data were handled anonymously, securely stored, and used exclusively for research purposes.

Study design

This retrospective cohort study was conducted using an online data collection form. The data were distributed among various co-authors to ensure a representative sample size. Statistical methods were employed to analyze the data and identify patterns or trends.

Study setting

The study was conducted in the Western Region of Saudi Arabia, specifically at KAUH in Jeddah, from January 2017 to August 2022.

Study population

The study targeted patients with ankle injuries who visited the Orthopedic Outpatient Department at KAUH in Jeddah. A total of 378 patients were initially recruited from the hospital records of the radiological department, with 215 meeting the inclusion and exclusion criteria and being included in the study.

Inclusion and exclusion criteria

This study included patients of any age or sex who visited our institution with ankle injuries and underwent MRI for the evaluation of ligamentous injuries and/or OCDs, as well as those who had undergone operative procedures. Patients were excluded if they met any of the following criteria: (1) ankle fracture, (2) any prior ankle surgery, (3) osteoarthritis of the ankle joint, (4) inflammatory arthritis, or (5) congenital deformity.

Sampling technique

To include individuals from the target population, we reviewed medical history, clinical examinations, MRI scans, and procedural reports from our hospital records.

Data collection

Data were collected using a simple questionnaire created with Google Forms (Google, Inc., Mountain View, CA, USA) and exported to Microsoft Excel 2016 (Microsoft Corp., Redmond, WA, USA), as detailed in the Appendix.

We assessed the presence of an ankle sprain or tear and OCDs based on MRI results and operative reports. Our previously prepared data sheet included demographic data, such as sex and age (see Appendix). To maintain patient confidentiality, names were not recorded; however, the medical record number was recorded for data tracking. We recorded the side of injury (right or left), name of the injured lateral ligament(s) (ATFL, PTFL, CFL, lateral talocalcaneal ligament, and/or syndesmosis), name of the injured medial ligament(s) (deltoid ligament and/or calcaneonavicular ligament), type of injury (partial or complete), and type of OCD (OCD of the talus and/or OCD of the tibial plafond).

Statistical analysis

Categorical data are expressed as frequencies (percentages), whereas continuous data are expressed as means and standard deviations. The chi-square test was used to calculate the correlations between variables. Statistical analyses were conducted using SPSS software (IBM Corp., Released 2012. IBM SPSS Statistics for Windows, Version 21.0, Armonk, NY: IBM Corp.).

## Results

Patient characteristics

In this study, 215 patients were recruited based on the inclusion criteria and categorized according to age, sex, presence of OCD, and type of ligamentous injury and its association with OCD in different areas.

**Figure 1:**
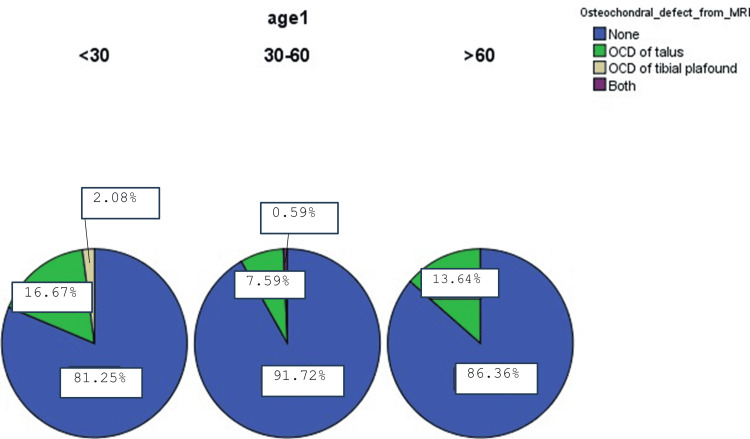
Osteochondral lesion (OCD) by age group MRI, magnetic resonance imaging

As illustrated in Figure 1, we categorized our results into three categories according to age group as follows: younger than 30 years (48 patients), 30-60 years (145 patients), and older than 60 years (22 patients). In the age group younger than 30 years, 39 patients (81.25%) did not have any OCD, eight patients (16.67%) had OCD of the talus, and one patient (2.08 %) had OCD of the tibial plafond. In the age group of 30-60 years, 133 patients (91.72%) did not have any OCD, 11 patients (7.59%) had OCD of the talus, and one patient (0.59%) had both OCD of the talus and tibial plafond. In the age group older than 60 years, 19 patients (86.36%) did not have any OCD and three (13.64%) had OCD of the talus. Overall, 24 patients of all ages had OCD on MRI.

**Figure 2:**
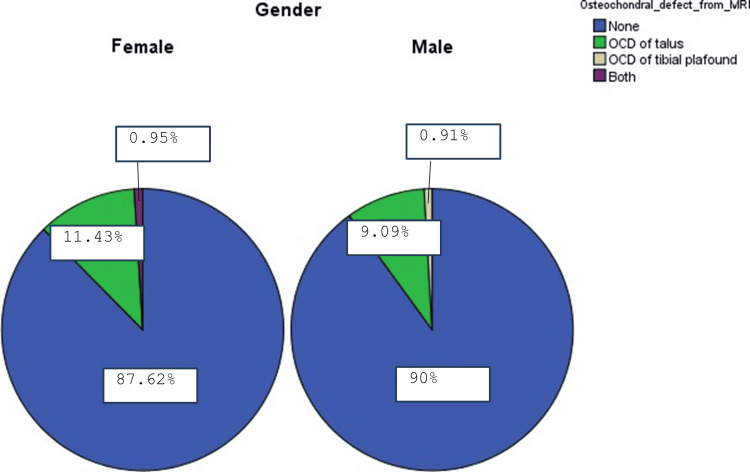
Osteochondral lesion (OCD) by sex MRI, magnetic resonance imaging

In addition, we divided patients by sex (105 females and 110 males), as shown in Figure 2. Among female participants, 92 (87.62%) did not have any OCD, 12 patients (11.43%) had OCD of the talus, and one patient (0.95%) had both OCD of the talus and tibial plafond. Among the male participants, 99 patients (90%) did not have any OCD, 10 patients (9.09%) had OCD of the talus, and one patient (0.91%) had OCD of the tibial plafond. However, a non-significant relationship was observed between sex and OCD of the talus and tibial plafond (p-value = 0.43 and 0.97, respectively).

Association of ligamentous injury with OCD of the talus

As shown in Table 1, 75 of the 215 patients had ATFL injuries; among them, eight had an associated OCD of the talus. These patients had ATFL injuries (three sprains, two partial tears, and three complete tears) and OCD of the talus. A non-significant relationship was noted between ATFL and OCD of the talus (P-value = 0.898).

**Table 1:** Association of anterior talofibular ligament (ATFL) injury with osteochondral defect (OCD) of the talus

		Anterior talofibular ligament				Total
		None	Sprain	Partial tear	Complete tear	
OCD of the talus	Absent	125	17	23	27	192
	Present	15	3	2	3	23
Total		140	20	25	30	215
P-value						0.898

However, as shown in Table 2, PTFL injury was prevalent in 15 out of the 215 patients, with four of these also having an associated OCD of the talus. The PTFL injuries included one sprain and three partial tears with OCD of the talus. The association between PTFL and OCD of the talus showed a significant relationship (P-value = 0.041). 

**Table 2:** Association of posterior talofibular ligament (PTFL) injury with osteochondral defect (OCD) of the talus

		Posterior talofibular ligament			Total
		None	Sprain	Partial tear	
OCD of the talus	Absent	181	6	5	192
	Present	19	1	3	23
Total		200	7	8	215
P-value					0.041

As shown in Table 3, among the 215 patients, 19 had deltoid ligament injury, with only three of these cases being associated with OCD of the talus. Their ligamentous injuries included a sprain and two partial tears with OCD of the talus. A non-significant relationship was observed between deltoid ligament injury and OCD of the talus (P-value = 0.273). 

**Table 3:** Association of deltoid ligament injury with osteochondral defect (OCD) of the talus

		Deltoid ligament				
		None	Sprain	Partial tear	Complete tear	Total
OCD of the talus	Absent	176	8	4	4	192
	Present	20	1	2	0	23
Total		196	9	6	4	215
P-value						0.273

Association between ligamentous injury and OCD of the tibia

Out of the 75 cases of ATFL injury, none had OCD of the tibia (Table 4). Only two patients had OCD of the tibia, with no ATFL injury. An insignificant association was noted between ATFL and OCD of the tibia (P-value = 0.782). 

**Table 4:** Association of anterior talofibular ligament (ATFL) injury with osteochondral defect (OCD) of the tibial plafond

		Anterior talofibular ligament				Total
		None	Sprain	Partial tear	Complete tear	
OCD the tibial plafond	Absent	138	20	25	30	213
	Present	2	0	0	0	2
Total		140	20	25	30	215
P-value						0.782

As shown in Table 5, PTFL injury was observed in 15 of 215 patients, with none of these cases being associated with OCD of the tibia. Isolated OCD of the tibia was observed in two cases. The association between PTFL and OCD of the tibia was insignificant (P-value = 0.927).

**Table 5:** Association of posterior talofibular ligament (PTFL) injury with osteochondral defect (OCD) of the tibial plafond

		Posterior talofibular ligament			Total
		None	Sprain	Partial tear	
OCD of the tibial plafond	Absent	198	7	8	213
	Present	2	0	0	2
Total		200	7	8	215
P-value					0.927

As shown in Table 6, 19 of the 215 patients had a deltoid ligament injury; however, none had associated OCD of the tibia. Only two patients had isolated OCD of the tibia. The association between the deltoid ligament and OCD of the tibia was insignificant (p-value = 0.996). 

**Table 6:** Association of deltoid ligament injury with osteochondral defect (OCD) of the tibial plafond

		Deltoid ligament				Total
		None	Sprain	Partial tear	Complete tear	
OCD of the tibial plafond	Absent	194	9	6	4	213
	Present	2	0	0	0	2
Total		196	9	6	4	215
P-value						0.996

As shown in Table 7, we sub-grouped our results according to sex and age for each ligamentous injury. ATFL injury was observed in 37 female participants (35.2%), and a similar incidence was seen in 38 male participants (34.6%) (p-value = 0.91). According to age group and type of injury, ATFL sprain and complete tear were the most common in those younger than 30 years at rates of 12.5% and 22.9%, respectively. In comparison, a partial tear was reported more commonly in the group aged 30-60 years (13.1%) than in the other groups.

**Table 7:** Ligamentous injuries and osteochondral defect (OCD) by age and sex

		Sex				Age (years)					
		Female		Male		<30		30–60		>60	
		Count	%	Count	%	Count	%	Count	%	Count	%
Anterior talofibular ligament	None	68	64.8	72	65.5	26	54.2	95	65.5	19	86.4
	Sprain	12	11.4	8	7.3	6	12.5	14	9.7	0	0.0
	Partial tear	13	12.4	12	10.9	5	10.4	19	13.1	1	4.5
	Complete tear	12	11.4	18	16.4	11	22.9	17	11.7	2	9.1
Posterior talofibular ligament	None	98	93.3	102	92.7	43	89.6	137	94.5	20	90.9
	Sprain	3	2.9	4	3.6	1	2.1	5	3.4	1	4.5
	Partial tear	4	3.8	4	3.6	4	8.3	3	2.1	1	4.5
	Complete tear	0	0.0	0	0.0	0	0.0	0	0.0	0	0.0
Deltoid ligament	None	99	94.3	97	88.2	41	85.4	134	92.4	21	95.5
	Sprain	3	2.9	6	5.5	5	10.4	3	2.1	1	4.5
	Partial tear	3	2.9	3	2.7	1	2.1	5	3.4	0	0.0
	Complete tear	0	0.0	4	3.6	1	2.1	3	2.1	0	0.0
OCD on MRI	None	92	87.6	99	90.0	39	81.3	133	91.7	19	86.4
	OCD of the talus	12	11.4	10	9.1	8	16.7	11	7.6	3	13.6
	OCD of the tibial plafond	0	0.0%	1	0.9%	1	2.1%	0	0.0%	0	0.0%
	Both	1	1.0%	0	0.0%	0	0.0%	1	0.7%	0	0.0%

PTFL injuries were reported in seven female participants (6.7%) and eight male participants (7.2%) (Table 7). According to different age groups and types of injury, PTFL sprain was more common in the age group older than 60 years of age (4.5%), whereas partial tear was observed more in the age group younger than 30 years (8.3%). No cases of PTFL complete tear were documented.

A slightly higher incidence of deltoid ligamentous injury was observed among male participants than among female participants (Table 7). This injury was observed in six female participants (5.8%) and 13 male participants (11.8%) (p-value = 0.11). Sprain injury of the deltoid ligament was observed more in the age group younger than 30 years (10.4%), whereas partial tears were more common in the age group 30-60 years (3.8%). A complete tear was equally observed in the age groups younger than 30 years and 30-60 years (both, 2.1%), with no reported cases in the age group older than 60 years.

## Discussion

Ankle injuries are among the most common musculoskeletal injuries, affecting individuals of all ages and activity levels [1,2]. Ankle sprains can range from mild to severe, with a spectrum of symptoms. While most ankle sprains are treated with conservative management, some can lead to chronic pain and instability, resulting in a higher risk of future injuries. Understanding the mechanisms of ankle sprain and its impact on the ligaments is crucial for developing effective prevention and treatment strategies.

In this study, we collected 215 recorded incidents of ankle ligamentous injuries. Our findings are consistent with those of another study that reported an almost equal sex distribution [14]. However, in contrast to the previous study noted that 63% of their patients were younger than 30 years of age, our study showed that approximately 67.5% of our patients were aged 30-60 years (145 patients), only 22.3% were younger than 30 years of age (48 patients), and the remaining 10.2% were older than 69 years of age (22 patients).

Regarding ATFL injury, the total number of injuries across all age groups was 75, accounting for 34.9% of all patients. This proportion was equally distributed between the sexes. Moreover, our study revealed that 66.7% (50 patients) of those patients with ATFL injuries were aged 30-60 years, 29.3% (22 patients) were younger than 30 years of age, and the remaining 4% (3 patients) were older than 60 years of age. These results suggest that ATFL injuries are most common in patients aged 30-60 years.

OCDs were found in 24 patients; however, only eight participants had concomitant ATFL injuries (three sprains, two partial tears, and three complete tears), which showed a non-significant association. None of the OCDs associated with ATFL injury were of the tibia; hence, all eight cases included OCDs of the talus. A study published in 2020 by Hadeed et al. with level 3 evidence supports our findings [15]. By contrast, a case series by Wang et al. in patients with ankle sprain and instability reported a significant association between lateral OCD of the talus and ATFL in the long term [13]. The differences between these studies are due to variations in the study design and methodology.

Regarding PTFL injury, the total number of injuries across all age groups was 15 among the 215 patients, accounting for 7%, which is lower than the incidence of ATFL injury due to differences in the mechanism of injury. According to the analysis of the 24 cases with OCD, only four had concomitant PTFL injury (one sprain and three partial tears), which showed a significant association between PTFL and OCD of the talus only. However, no recent studies have discussed the association between PTFL and OCD. This could be explained by the fact that the PTFL is usually injured in more severe ankle sprains than other ligaments, and the incidence of PTFL injury is less than that of other ligament injuries [13].

The deltoid ligament is a crucial medial structure that helps to restrict ankle external rotation and eversion, and there is potential for deltoid ligament injury to be used as a predictor of future instability [16]. Furthermore, concerning deltoid ligament injuries, our study found that lateral ligament injuries were the most common type of ligamentous injury. Simultaneously, medial ligament injuries could also occur depending on the mechanism. The total number of deltoid ligament injuries across all age groups was 19, accounting for 8.8% of the study population. Among these, only three patients had concomitant OCD of the talus, and none of them had OCD of the tibia. Khor conducted a study on 64 patients with ankle MRI and reported that an average of 27% of deltoid ligament sprains occurred. The frequencies of deltoid damage were 35% in the patient group with MRI evidence of lateral ligament injury and 50% in patients with lateral ligament injuries combined with other injuries [16]. However, the analysis of the association between deltoid ligamentous injury and OCDs in our study was insignificant.

Moreover, the MRI reports of the 24 cases with OCD showed that the majority were located at the talus (22 patients); a single patient had OCD at the tibia, and another patient had a combined injury of both the tibia and talus. This finding was almost equally distributed across both sexes, with the majority of the affected patients (12 cases) aged 30-60 years. However, in our study, there is non-significant relationship between age and OCD. By contrast, another study with a sample size of 1,169 patients found a significant association between OCD and older male patients [13].

This study has some limitations. First, the MRI reports reviewed for the 215 patients were not assessed by a single radiologist, which exposed the data to minimal interobserver variability in the interpretation of MRI imaging results. Second, while the cofactors that might lead to an ankle ligamentous injury, including peroneal ligament dysfunction, concurrent osteoarthritis of the ankle, and active rheumatoid arthritis in the ankle joint, were excluded as much as possible, these factors cannot be ruled out.

## Conclusions

In the context of OCDs and ankle ligament injuries among the Saudi Arabian population, our study found that the most common OCD, by age and sex, was OCD of the talus. Conversely, the most frequently injured ligament was the ATFL. However, no significant relationship was found between OCD and ATFL injuries. Notably, a significant relationship was observed between PTFL injuries and OCD of the talus. Early diagnosis of these injuries is crucial for better outcomes, as concomitant injuries such as PTFL damage and OCD of the talus should be considered during patient assessment and operative management to prevent further complications.

## Figures and Tables

**Figure 3:**
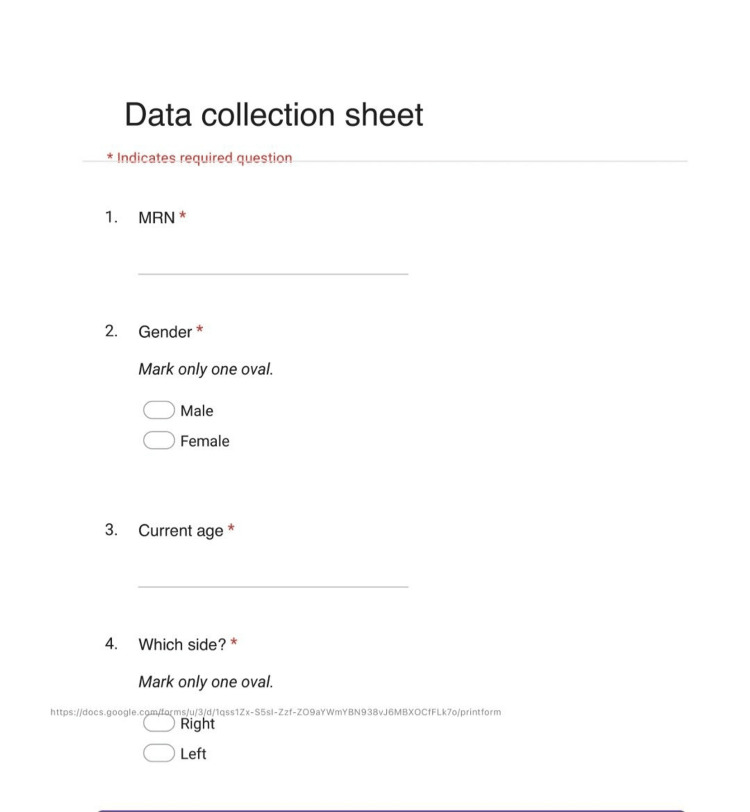
Data sheet

**Figure 4:**
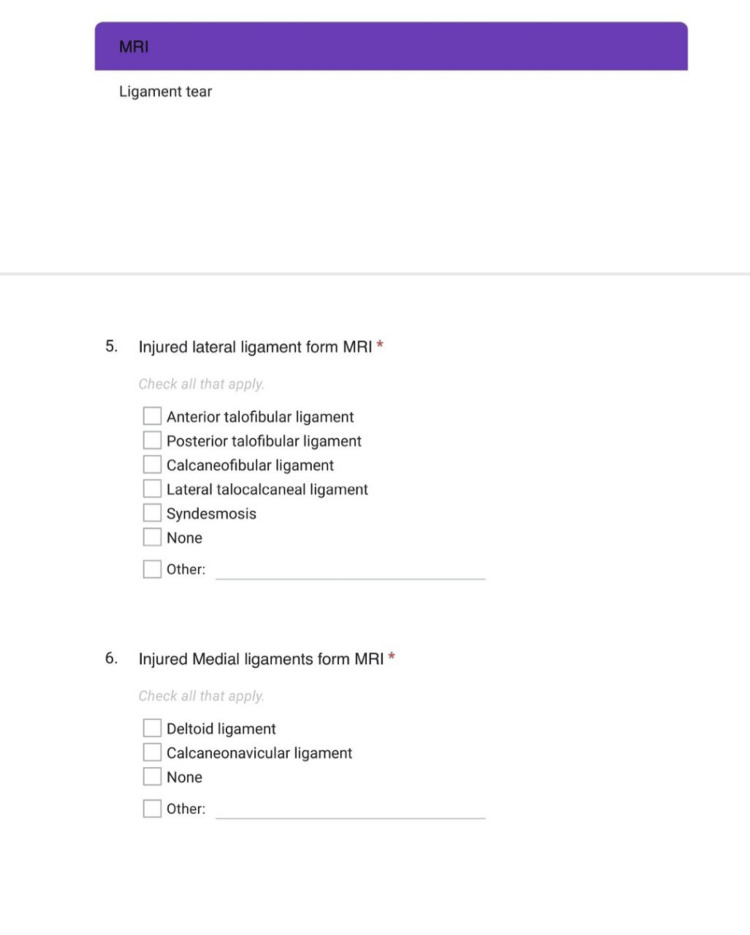
Data sheet

**Figure 5:**
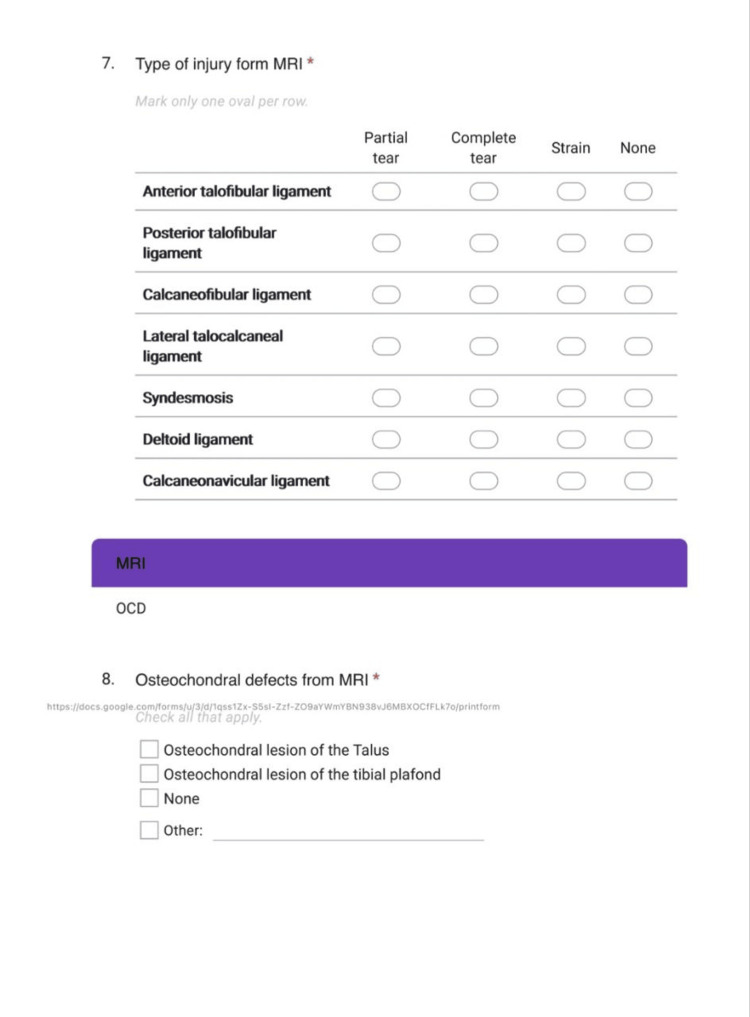
Data sheet
